# Effect of Size of Coarse Aggregate on Mechanical Properties of Metakaolin-Based Geopolymer Concrete and Ordinary Concrete

**DOI:** 10.3390/ma14123316

**Published:** 2021-06-15

**Authors:** Hamed Fazli, Dongming Yan, Yajun Zhang, Qiang Zeng

**Affiliations:** College of Civil Engineering and Architecture, Zhejiang University, Hangzhou 310058, China; hamedfazli@zju.edu.cn (H.F.); yj_zhang@163.com (Y.Z.); cengq14@zju.edu.cn (Q.Z.)

**Keywords:** geopolymer concrete, OPC concrete, coarse aggregate size, metakaolin, microstructure

## Abstract

Geopolymer binders are a promising alternative to ordinary Portland cement (OPC) because they can significantly reduce CO_2_ emissions. However, to apply geopolymer in concrete, it is critical to understand the compatibility between the coarse aggregate and the geopolymer binder. Experimental studies were conducted to explore the effect of the size of the coarse aggregate on the mechanical properties and microstructure of a metakaolin-based geopolymer (MKGP) concrete and ordinary concrete. Three coarse aggregate size grades (5–10 mm, 10–16 mm, and 16–20 mm) were adopted to prepare the specimens. The microstructure of the concretes was investigated with scanning electron microscopy/energy-dispersive X-ray spectroscopy (SEM/EDS) and mercury intrusion porosimetry (MIP). Results showed an opposite coarse aggregate size effect between OPC and MKGP specimens in terms of compressive strength. SEM/EDS analysis indicated that the MKGP concrete has a weaker microstructure compared to OPC concrete induced by a higher porosity. The differences in mechanical properties and pore structure between the MKGP and OPC concrete are attributed to the greatly differing shrinkages triggered by the large surface area and penny-shaped particles of metakaolin. The findings in this work help tailor the mechanical properties and microstructure of MKGP concrete for future engineering applications.

## 1. Introduction

Ordinary Portland cement (OPC) concrete is the most widely used and essential material for most construction industries. Yet, its production leads to several environmental issues such as the depletion of natural raw materials including limestone and clay as well as air pollution [1]. Moreover, cement production has a high level of CO_2_ emission [2], close to 8% of the global CO_2_, and possibly adversely affects climate change and global warming [3,4]. These issues led the researchers to explore ecological green concrete materials such as geopolymer concrete (GPC). Geopolymer cement was introduced by Davidovits et al. [5] and it elicited a growing interest due to its attractive properties, including higher resistance to chemical attacks [6,7], resistance to freeze-thaw cycles [8], and excellent resistance to elevated temperatures [9]. Geopolymer is an inorganic polymer formed by mixing alumina (Al_2_O_3_) with alkali liquids. Research on the use of fly ash, slag, silica fume, and metakaolin, as a source of aluminosilicate, has been increased [10]. Metakaolin (MK) is a natural source of alumina with some unique characteristics. It can gain a high early strength without any water curing [11]. MK improves the concrete strength and durability significantly and its production can be well controlled to achieve high purity and high pozzolanic reactivity [12].

Concrete is a quasi-brittle material. It is important to study mechanical parameters comprehensively. Mechanical parameters include, in particular, compressive strength, tensile strength, modulus of elasticity, and fracture energy [13]. The performance of composite materials such as concrete highly depends on the interaction between the constituents and their chemical and physical properties. Concrete is a heterogeneous material consisting of a three-phase system: cement paste, coarse or fine aggregate, and the ITZ between the coarse aggregate and matrix [14]. The coarse aggregate has a key role in determining the mechanical behavior of concrete as it occupies about 70% of the concrete volume [15]. Nili and Ehsani [16] noted that the coarse aggregate, cement paste, and the interfacial zone (ITZ) between them significantly affect the mechanical properties of concrete. Extensive research has been done on the effect of the coarse aggregate on the properties of OPC concrete. The previous studies showed that the size of the coarse aggregate is a critical factor that influences the development of the ITZ area and subsequent formation and propagation of microcracks [17,18]. The size of the coarse aggregate is a crucial factor that could impact the mechanical properties and durability of OPC concrete [19,20]. Ćosić, Korat, Ducman and Netinger [19] found that smaller-sized coarse aggregates can result in higher flexural strength in the OPC concrete. Contrary to the Zhong and Wille [20] findings, the compressive strength of OPC concrete increases with the increase of the coarse aggregate size, as reported by Yu et al. [21].

However, the majority of the early studies on the geopolymer focused on the mortar and paste. Numerous investigations have been conducted on the influence of various parameters on geopolymer concrete (GPC), such as alkali precursor (such as fly ash) ratio, Si/Al ratio, alkali concentration, and the properties of the coarse aggregate [22,23]. The physical and mechanical properties of fly ash-based geopolymer concrete, compared to those of Portland cement concrete were studied by [24]. It was revealed that the ratio of binder to aggregates somehow has a significant effect on the properties of geopolymer concrete. According to the findings, the coarse aggregate content effect in the case of GPC varies widely [25,26]. Even though there are studies that have been conducted on the influence of various parameters on the geopolymer, the size effect of the coarse aggregate on the GPC remains uncertain, while its importance and influence on the mechanical properties and durability of OPC concrete are well accepted. Moreover, a highly robust and reliable mix design procedure suitable for GPC is yet to be established. Therefore, the present study focuses on the influence of the size of the coarse aggregate on the metakaolin-based geopolymer (MKGP) concrete.

Even though intensive studies are performed on various fronts, for the construction industry use of MKGP concrete, a lot of research, regulation, and systemization demands to be accomplished. The above-mentioned studies have shown that the microstructure of the OPC concrete varies depending on the size of the coarse aggregate, which influenced its mechanical properties. Besides, the previous studies on GPC concrete mainly focused on the coarse aggregate content and properties rather than the size. As these properties considerably affect the OPC concrete performance and durability, therefore, it is imperative to extend the literature to study the effect of the size of the coarse aggregate on the MKGP concrete strength and microstructure where, to the best of our knowledge, it has not been investigated. It is still a matter of debate, and much work on the investigation of the effect of the coarse aggregate size on the mechanical properties and microstructure of MKGP concrete remains to be performed. Although most of the previous studies on the mechanical behavior of geopolymer concrete were conducted under elevated temperature conditions, this study reports the findings obtained under ambient temperature, which is the practical application condition.

An experimental study was therefore conducted to investigate the effect of the coarse aggregate size on the mechanical properties of the MKGP concrete. The failure mode, compressive strength, splitting tensile strength, scanning electron microscopy/energy-dispersive X-ray spectroscopy (SEM/EDS) analysis, and pore structure characterizations were employed to evaluate the MKGP concrete strength performance. The results were compared with the OPC concrete to present the implications for potential engineering applications. These findings help better understand the effect of the coarse aggregate size on GPC properties.

## 2. Experimental Procedures

### 2.1. Materials

**Figure 1:**
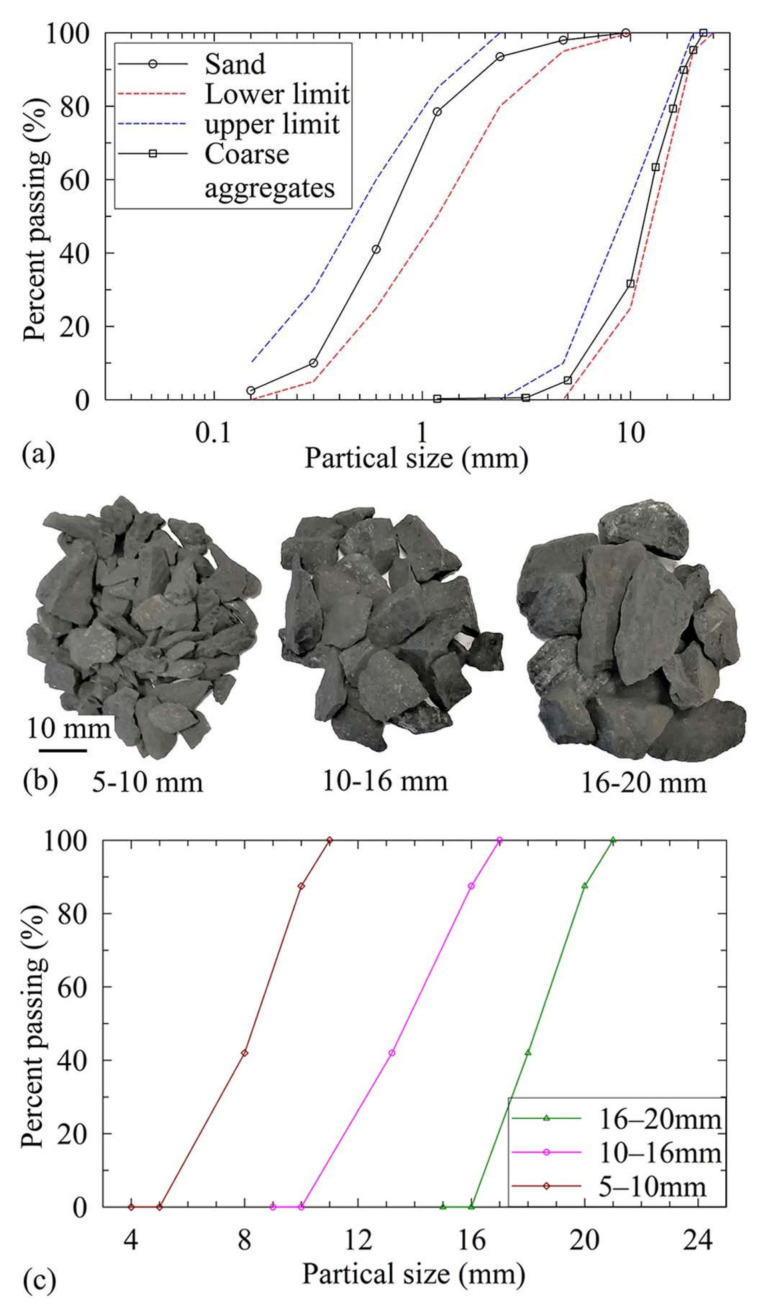
Coarse aggregate and sand used in this study: (**a**) particle size distribution curve for the coarse aggregate and sand; (**b**) three range of the sizes of the coarse aggregates; (**c**) the grain size distribution curve for the three size ranges of the coarse aggregates.

Typical OPC concrete specimens were prepared using the cement of CEM I 42.5 N [27]. The industrial-grade metakaolin (MK) was acquired from BASF MetaMax. Table 1 presents the chemical compositions of OPC and MK obtained from X-ray fluorescence (XRF) tests. The XRF spectrum was recorded using a Malvern PANalytical Epsilon 1 spectrometer (Malvern PANalytical, Almelo, Netherlands). XRF spectroscopy is a non-destructive and accurate technique used to identify material composition and sample preparation is mostly not required. The chemical analysis revealed that the sum of SiO_2_, Al_2_O_3,_ and Fe_2_O_3_ content was 95.46%. This is above the minimum value of 70% as required by ASTM C618 [28]. Furthermore, the MgO content was within 5%. Sodium silicate (water glass, WG) and a pellet sodium hydrate were used to prepare the alkaline activator solution. The chemical composition of the water glass is reported in Table 2. The pellet sodium hydrate was AR (analytical reagent) level with a purity of 96%. The dry river sand and limestone coarse aggregate were used for all the specimens in this study. Figure 1a shows the grain size distribution of the sand and coarse aggregate. The fineness modulus of the sand and coarse aggregate was 2.8 and 4.34, respectively. The water absorption capacity of the coarse aggregate was 0.9% and the density was 2620 kg/m^3^. The standard sieves were employed to prepare the three groups of coarse aggregate, as shown in Figure 1b. Their particle size distribution curves are plotted in Figure 1c. The coarse aggregate with three size ranges (5–10 mm, 10–16 mm, and 16–20 mm) and a fineness modulus of 2.73 were used to prepare the specimens.

### 2.2. Test Specimen Preparation

The concrete mixes with a grade of 40 MPa compressive strength at 28 days were prepared based on 5–10 mm coarse aggregate size, as summarized in Table 3. Before casting, to reduce the water absorption effect on the test results, the coarse aggregates were immersed in water for 24 h, after which their surface water was dried by placing them over a large sieve for 2 h and leaving water to evaporate [29]. The coarse aggregate and sand were first dry-mixed for two minutes. Then, for casting the MKGP concrete, the alkaline activator solution and water were added gradually, and mixing continued for five minutes. The alkaline activator solution was prepared at least 24 h before the synthesis of the geopolymer by mixing a liquid sodium silicate (water glass, WG) and a pellet sodium hydrate according to the mixture design. For the OPC concrete casting, water was gradually added after a dry mix of the coarse aggregate, sand, and cement, and mixing continued for five minutes. The fresh MKGP and OPC concrete were cast into respective molds in three layers. Each layer was compacted 25 times with a standard steel rod according to the procedure reported in BS EN 12390-2 [30].

Three concrete cubes with the dimensions of 150 mm × 150 mm × 150 mm and 100 mm × 100 mm × 100 mm were made for each group to measure the splitting tensile strength ($f_{t}$) and compressive strength ($f_{cu}$), according to BS EN 12390-6 [31] and BS EN 12390-3 [32], respectively. All the specimens of OPC and MKGP concrete were wrapped with plastic film to prevent water loss and demolded after 48 h, then stored in a curing room at 20–25 °C and 90–95% RH until the testing day. The results of the mechanical tests and their standard deviations (SDs) are presented in Table 4. A specimen label was assigned to each cube as X-Y-Z. The first term (X) refers to the material type, which is either the OPC or the MKGP. The second term (Y) denoted as 1, 2, and 3 consists of the coarse aggregate size range of 5–10 mm, 10–16 mm, and 16–20 mm, respectively. The last term (Z) refers to the number of specimens.

**Figure 2:**
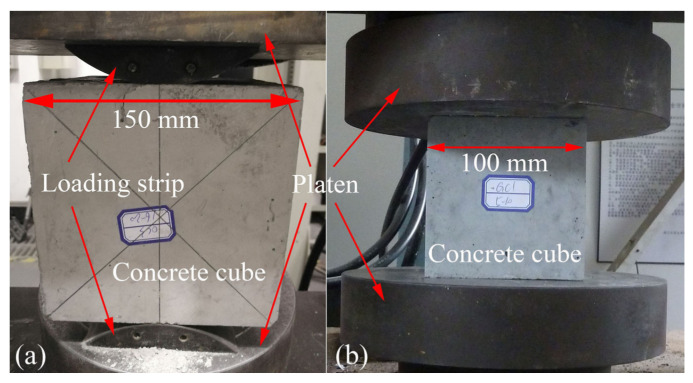
Test set-up for (**a**) splitting tensile test; (**b**) compression test.

In this study, 18 specimens were tested after 28 days of curing for compressive strength and splitting tensile strength. The loading rate was 0.3 MPa/s. Figure 2 presents the dimensions and loading of the specimens.

### 2.3. Microstructural Analysis

Microstructural investigations were conducted on the fractured surfaces of both OPC and MKGP concrete specimens. The microanalytical technique SEM/EDS analysis was performed to study the interfacial morphology between the paste and coarse aggregate. The Quanta FEG650 (FEI, Hillsboro, OR, USA) SEM/EDS instrument was used at the 5- or 8- or 10-kV accelerating volt (FEI) model. The interfacial regions between the paste and the coarse aggregate were studied through microstructural investigations of the ITZ area in the specimens of the OPC and MKGP concrete considering the size of the coarse aggregate. Moreover, the mercury intrusion porosimeter (MIP; Micromeritics AutoPoreIV9500, Norcross, GA, USA) test was employed to determine the porosity and pore size distribution of the tested specimens.

## 3. Results and Discussion

### 3.1. Failure Behavior

**Figure 3:**
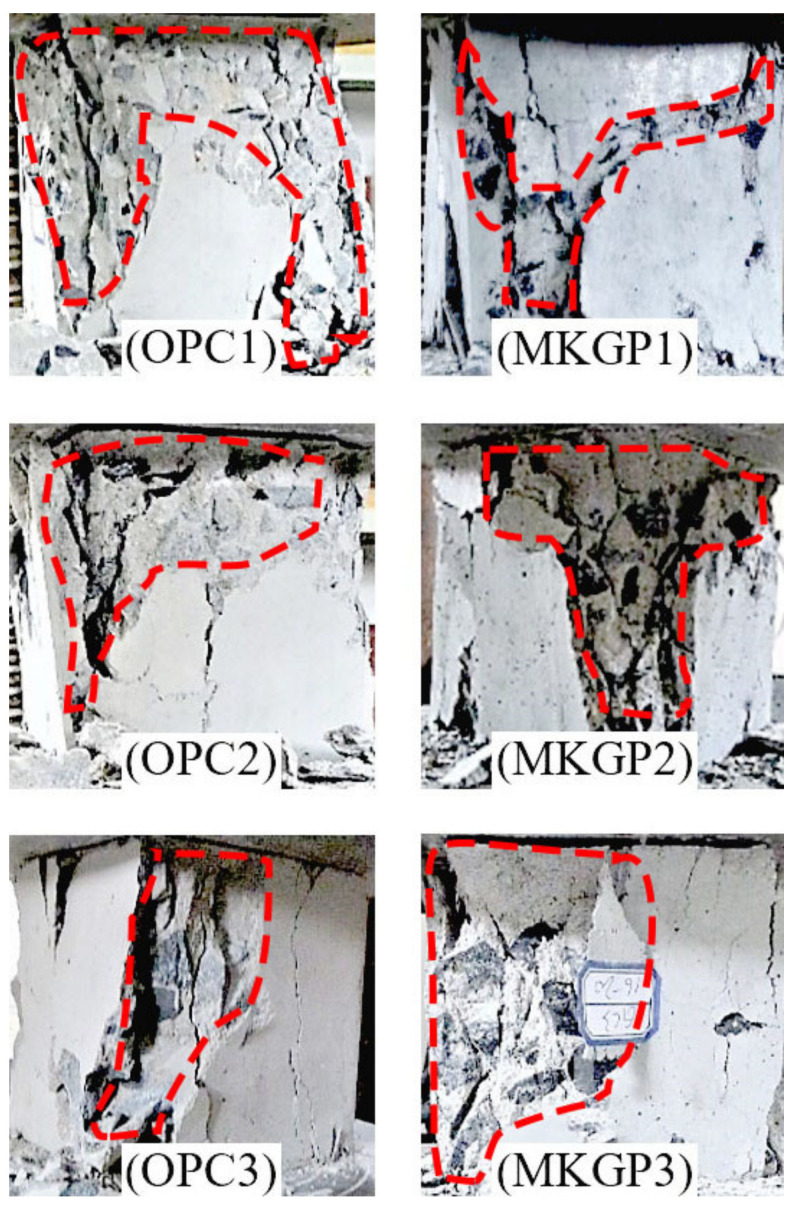
OPC (**left**) and MKGP (**right**) concrete cube specimens after compression test.

Figure 3 illustrates the failure types of the specimens after the compression test. OPC concrete fractured specimens showed that, as the size of the coarse aggregate increased, the observed spalled material relatively decreased, which could be attributed to the increase of the compressive strength of the specimens. In contrast, the MKGP concrete specimens yielded a different behavior. As the size of coarse aggregate increased, a greater extent of crushing was observed, which could be due to the decrease of the compressive strength. Although MKGP specimens exhibit more fracture and brittle behavior than the OPC specimens during the compression test, in general, the MKGP concrete specimens showed similar cracking and failure patterns compared to that of the OPC concrete specimens [33]. Tested specimens displayed cracking because of splitting along the height of the cubes due to the development of tensile stresses. The debris from compression tests proved that the quality of the coarse aggregate was desirable, as the crack formed in the cement paste and not in the coarse aggregate.

### 3.2. Compressive Strength

**Figure 4:**
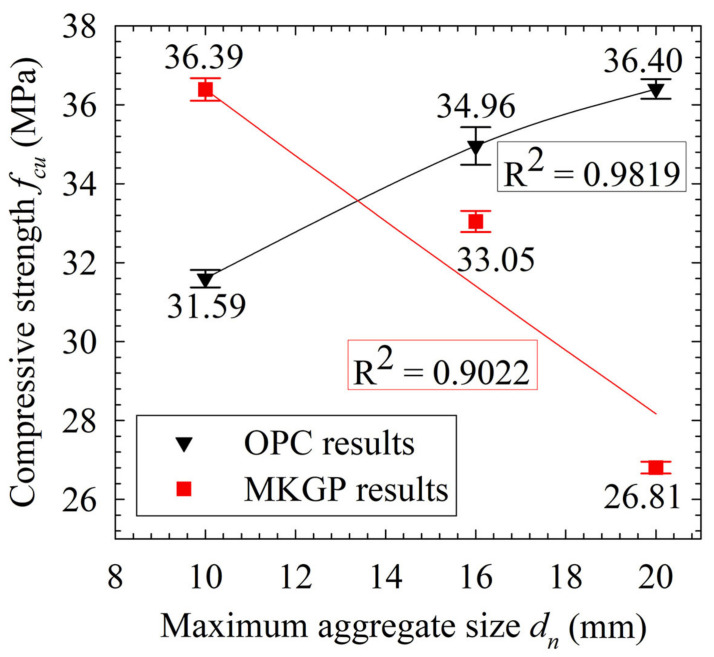
Experimental results of compressive strength.

The average compressive strength test results of OPC concrete and MKGP concrete are presented in Table 4 and depicted in Figure 4. As the coarse aggregate size increased, the compressive strength of the OPC concrete increased. This trend ranged from 31.59 MPa for the 5–10 mm to 36.40 MPa for the 16–20 mm coarse aggregate size. The results are consistent with the study by Yuan et al. [34] in which the compressive strength increases when the aggregate size increases. With the same weight of the coarse aggregate in OPC specimens, the larger coarse aggregate leads to a less specific surface area, so it is surrounded by a thicker OPC paste. Consequently, the paste between the larger coarse aggregate would have better quality and fewer microcracks [35], which yielded a higher compressive strength.

The MKGP concrete results yielded a decrease in compressive strength values as the coarse aggregate size increased. The results are consistent with the study by Guades [36], showing the effect of aggregate size variation on the compressive strength of the GPC under ambient temperature. The considerable decrease of compressive strength observed in specimens prepared with 16–20 mm size aggregates implies that the coarse aggregate size could significantly affect the strength of the MKGP concrete. Unlike the OPC specimens, the large volume of the paste around the coarse aggregate reduces the compressive strength. It could contribute to the higher shrinkage of the MKGP paste compared to the OPC concrete, resulting in a highly porous paste of the MKGP concrete, which will be discussed further in Section 4.

### 3.3. Splitting Tensile Strength Test Results

**Figure 5:**
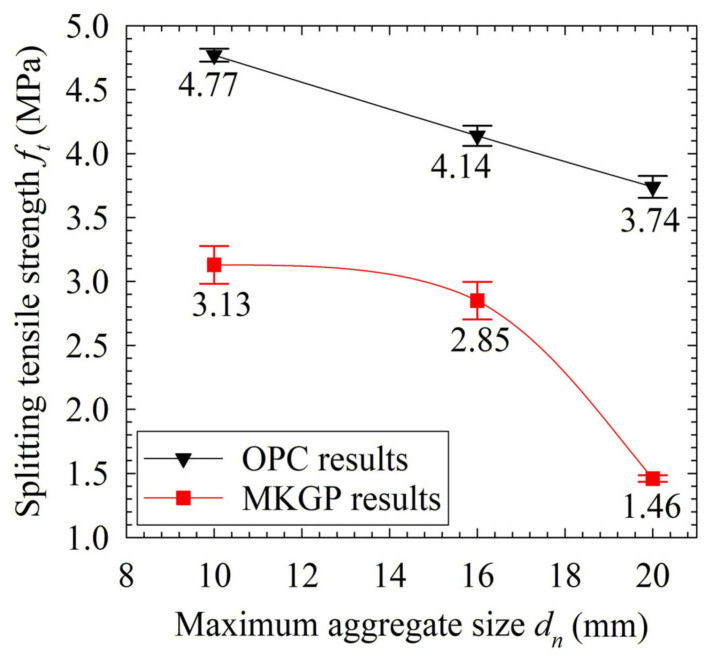
Experimental results of compressive strength.

Table 4 and Figure 5 present the splitting tensile strength test results of the OPC and MKGP concrete. These results illustrate that, despite the increase of the aggregate size, the splitting tensile strength decreased which is consistent with the previous studies [37]. It also implicates that a larger coarse aggregate cannot enhance the splitting tensile strength of concretes.

The larger volume of the paste between the larger coarse aggregate leads to a more pronounced difference between the elastic modulus of the coarse aggregate and paste as a result of the increased coarse aggregate volume relative to the specimen volume [38]. Consequently, it increases the stress concentration and results in more microcracks near the coarse aggregate. It, therefore, yields a higher reduction in the splitting tensile strength of concrete with a lower w/c ratio of 0.4, as reported by Akçaoğlu, Tokyay and Çelik [38]. They revealed that the interfacial bond is a critical factor for tensile strength compared to its role in compressive strength. 

Physically, the small surface area could result in the lower development of the gel bond, and consequently, increases the shrinkage cracks near the coarse aggregate. Moreover, the possible internal bleeding underside of a larger coarse aggregate could contribute to decreasing the splitting tensile strength by reducing the interfacial bond strength inside the concrete.

Results show that the decrease of the $f_{t}$ value in MKGP is more rapid than that of OPC concrete. It could attribute to the higher shrinkage cracks of the MKGP paste compared to that of OPC. Furthermore, it may be attributed to the weaker microstructure of the MKGP paste compared to that of the OPC specimens, as discussed in Section 3.5.1.

Mechanical test results manifest the different microstructure behaviors of the MKGP concrete compared with the OPC concrete. Moreover, the development of the interfacial bond may vary in the MKGP concrete with different coarse aggregate sizes compared to the OPC concrete. This effect, however, is more pronounced in OPC concrete, probably due to relatively better compatibility between the OPC and coarse aggregate phases as compared to MKGP.

### 3.4. Proposed Empirical Models

Compressive strength and splitting tensile strength are essential material properties of the concrete to determine the design purposes and structural behavior of the concrete member. In addition, the tensile strength of the concrete could be estimated from its compressive strength. In this study, new empirical equations are therefore proposed to link the compressive and splitting tensile strengths of MKGP concrete and OPC concrete by considering the coarse aggregate size effect.

#### 3.4.1. Effect on the OPC Concrete

To predict the compressive strength of concrete by considering the coarse aggregate size effect, Jiang et al. [39] proposed a model based on Bazant’s law of size effect and the calibrated model by Kim et al. [40].

(1) $$f_{c}=f_{c}^{\prime }\cdot \delta (d_{\max},h,d_{a}^{m})$$

(2) $$\delta (d_{\max},h,d_{a}^{m})=\alpha +\frac{B}{\sqrt{1+\frac{d_{\max}}{\lambda_{0}d_{a}^{m}}\left(\frac{h}{d}-\beta \right)}}$$

where $f_{c}$ and $f_{c}^{\prime }$ are the actual cylinder compressive strength of the concrete specimen considering the size effect and the strength of the concrete specimen of standard size (designed compressive strength = 40 MPa), respectively. α, B, $\lambda_{0}$ and β are the coefficients that can be determined via experimental results. $d_{\max}$ is the maximum coarse aggregate size of concrete. h and d define the specimen size. The regression analysis of Kim, Yi, Park and Eo [40] yielded $d_{a}^{m}\approx 1$. Therefore, the cylindrical compressive strength of concrete considering the coarse aggregate size can be expressed as follows:

(3) $$f_{c}=\alpha f_{c}^{\prime }+\frac{Bf_{c}^{\prime }}{\sqrt{1+\frac{d_{\max}}{\lambda_{0}}\left(\frac{h}{d}-\beta \right)}}$$

The designed compressive strength ($f_{c}^{\prime }$), in this study, is 40 MPa, and the h/d ratio is equal to 1, as the tested specimens were concrete cubes. Moreover, the measured cube compressive strength can be converted to the cylinder compressive strength via $0.8f_{c}^{\prime }$. Equation (3) for the tested specimens can therefore be expressed as follows:

(4) $$f_{c}=0.8\times \left(\alpha f_{c}^{\prime }+\frac{Bf_{cu}^{\prime }}{\sqrt{1+\frac{d_{\max}}{\lambda_{0}}\left(\frac{h}{d}-\beta \right)}}\right)$$

The measured experimental cube compressive strength (Table 4) and Equation (4) were used to determine the relationship between the compressive strength and the maximum coarse aggregate size, as shown in Figure 4. A Levenberg–Marquardt algorithm in Matlab [41], a curve fitting toolbox, was used to establish the fitting parameters. It yields that α, B, $\lambda_{0}$ and β are equal to 1.570, −1.351, 2.171, and 1.052, respectively. The splitting tensile strength of concrete ($f_{t}$) can be estimated based on its compressive strength, as reported by ACI 318-14 [42] and CEB-FIP [43]:

(5) $$f_{t}=\varphi {\left(f_{c}\right)}^{c}$$

**Figure 6:**
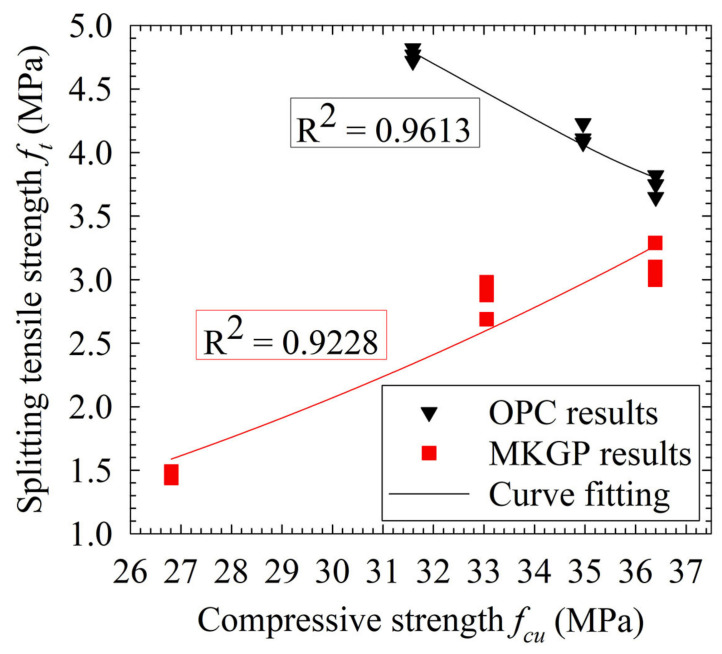
Relationship between Equation (5) and experimental results.

Figure 6 depicts that the experiments’ splitting tensile strength results compare well with the predicted compressive strength results. The obtained fitting parameters yielded that φ and c are equal to 1334 and −1.63, respectively.

#### 3.4.2. Effect on the MKGP Concrete

Figure 4 also shows the effect of the coarse aggregate size on the compressive strength of the MKGP concrete. The interesting point is the opposite effect of the larger size of the coarse aggregate on the compressive strength of MKGP specimens compared with the OPC specimens. Therefore, Equation (4) was employed to describe the relationship between the MKGP compressive strength and the maximum coarse aggregate size. After fitting the analysis of the experimental results, the coefficients α, B, $\lambda_{0}$ and β are determined as −17.43, 18.83, 2.336, and 1.993, respectively. The calibrated model is employed to predict the compressive strength, as given in Table 5. Comparing the experimental results ($f_{cu}$) with the calibrated values ($f_{c}$) revealed that the predicted result matches well with the experimental results, as its mean value and the standard deviation (SD) are 0.98 and 0.041, respectively.

From an experimental point of view, measuring the splitting tensile strength directly from concrete specimens is not always easy. The relationship between splitting tensile strength and compressive strength of tested MKGP concrete specimens are therefore provided (Figure 6) to avoid the direct measurements which are demanding and time-consuming. According to Equation (5), the following relationship can be established:

(6) $$f_{tg}=0.0006775{\left(f_{cg}\right)}^{2.359}$$

where $f_{tg}$ and $f_{cg}$ are the splitting tensile strength and cube compressive strength of the geopolymer, respectively. The proposed model is employed to predict the splitting tensile strength, as given in Table 5. Comparing the experimental results ($f_{cu}$) with the model values ($f_{c}$) yielded acceptable prediction values, as its mean value and the standard deviation (SD) are 0.93 and 0.077, respectively.

### 3.5. Microstructure Analysis

#### 3.5.1. SEM Observation and EDS Analysis

**Figure 7:**
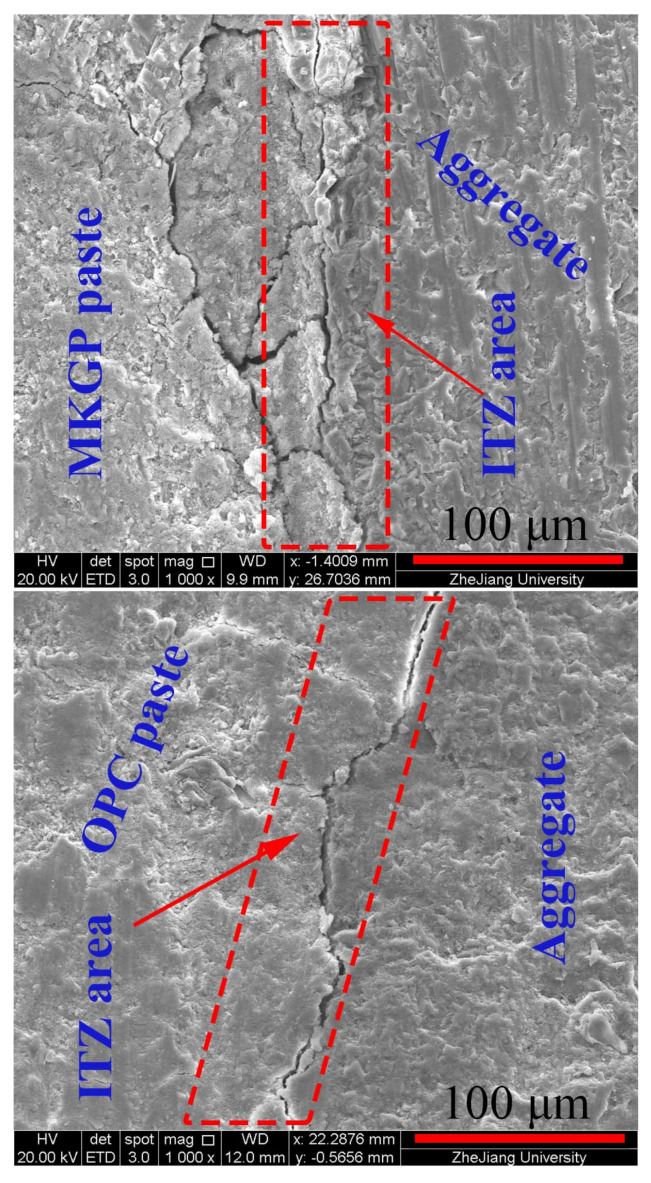
Microstructure of the MKGP3 and the OPC3.

**Figure 8:**
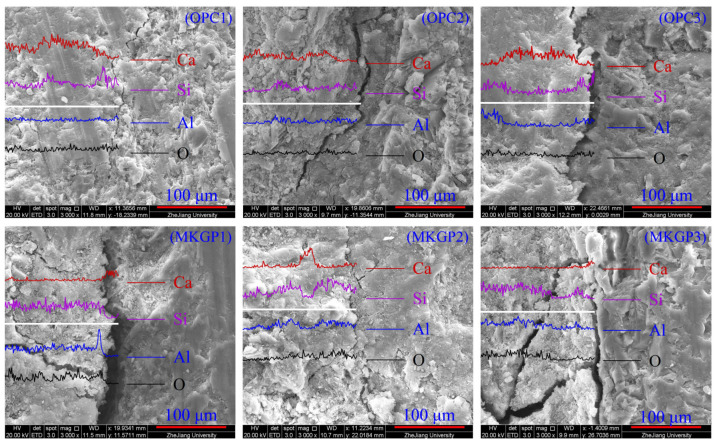
EDS Line scan analysis and SEM images of the OPC concrete and the MKGP concrete.

SEM/EDS investigations were conducted on the fracture surfaces of the specimens. Figure 7 shows the identification of the coarse aggregate zone, paste zone, and ITZ area in the SEM images. The line scan analysis of SEM/EDS presents the element intensity of the OPC and MKGP concrete specimens, as shown in Figure 8. The structure of the samples illustrated in Figure 8 is similar to Figure 7. The data were obtained in 10 µm intervals from the paste to the coarse aggregate surface to determine the characteristics of the microstructure of the tested specimens.

The microstructure of the ITZ area around the different coarse aggregate sizes in both OPC and MKGP concrete was investigated, as shown in Figure 8. SEM observations showed that the ITZ area is looser than other parts of the paste. It manifests the existence of a more porous zone in MKGP specimens, which would be the weaker part of the sample. It may be attributed to the formation mechanism of ITZ for OPC and MKGP, which may not be the same. Microstructure analysis revealed that the coarse aggregate sizes investigated in this study do not significantly affect the ITZ area. It shows that the MKGP paste resulted in a weaker microstructure compared to that of the OPC specimens. 

In general, increasing the coarse aggregate size did not exhibit any considerable influence on the EDS analysis in this study, as shown in Figure 8. Along the normal direction of the ITZ, the 255 point data were obtained at 127.5 μm (0.5 μm) intervals from the aggregate to paste. The results, however, revealed the presence of a C-S-H phase (calcium-silicate-hydrate) because of the hydration of pure OPC. The three distinct phases that compose the cementitious OPC matrix were as follows:

- Portlandite, which the brittle Ca(OH)_2_;
- Hygrated Ca silicate;
- Hydrated Ca aluminate.

The Ca atoms’ intensity decreased from a concrete paste into the coarse aggregate, indicating the percentage of CH particles on the paste side is mainly responsible for the porosity in the ITZ [44]. Si and Al are the main components of the N-A-S-H gel in the metakaolin geopolymer [45], which were observed in the paste part of MKGP concrete specimens. The presence of Al and Si leads to the formation of N-A-S-H, and together with Ca could contribute to a cohesive mix during the concrete mixing, which is also supported by visual observations during specimen preparation. The hydration product has different Ca/Si and Al/Si ratios. EDS analysis of OPC samples showed the decrease of Ca atoms and Si atoms with the size of the coarse aggregate. The decrease in Ca/Si ratio may attribute to the porous ITZ area. Moreover, in MKGP samples, the Si/Al ratio increased with the size of the coarse aggregate indicating the less dense structure.

#### 3.5.2. Pore Structure Analysis Using MIP

**Figure 9:**
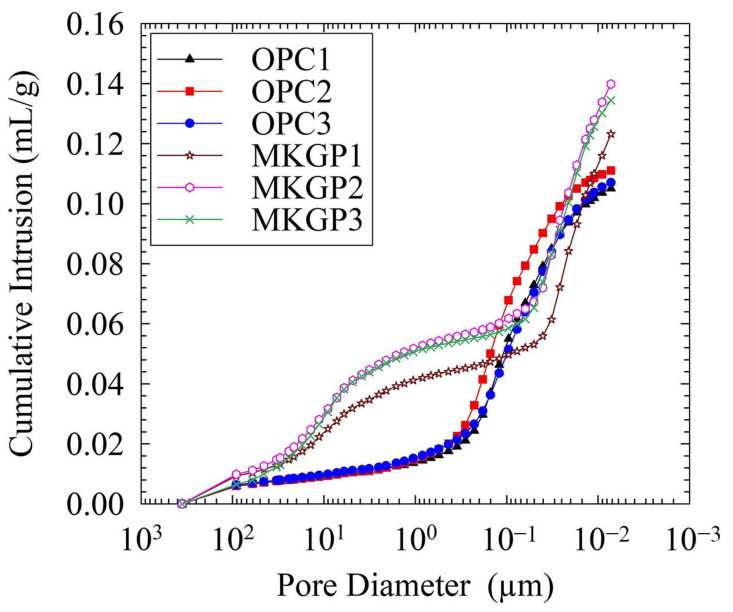
Cumulative intruded pore volume vs. pore diameter for OPC and MKGP specimens.

**Figure 10:**
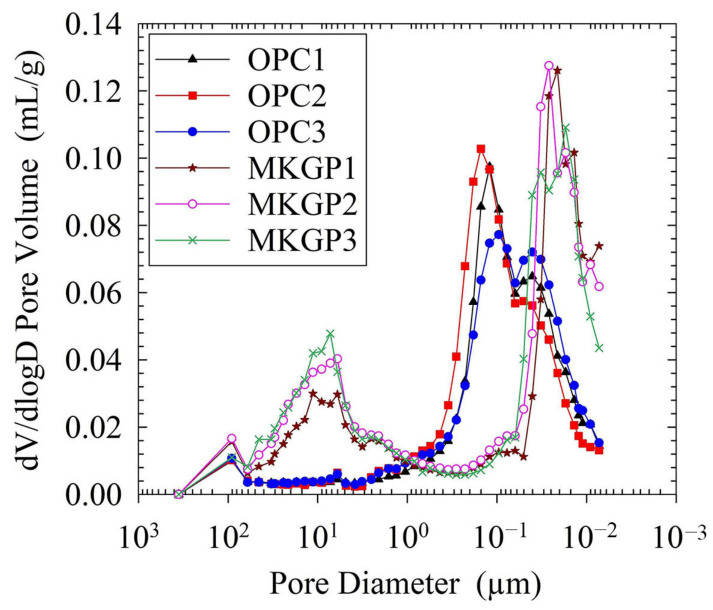
Differential pore size distribution curves of the OPC and MKGP specimens.

The porosity and pore size distribution of the OPC and MKGP concrete specimens were obtained using MIP tests. Figure 9 and Figure 10 show the pore size distribution of the concrete specimens. The test results were obtained from the crushed concrete specimens weighing approximately 1.5 g. Figure 9 shows the variations in cumulative intruded pore volumes plotted as a function of pore size diameters. The results show that the OPC specimens’ intrusion curves are almost similar, as well as the MKGP specimen curves. The MKGP intrusion curves, however, exhibit significant differences when the diameter of the pores is between 0.2 µm and 20 µm. It yields the differences in pore structure between MKGP specimens and OPC specimens. Moreover, the MKGP curves show that an increase of the coarse aggregate size slightly changes the pore size distribution, especially when the diameter of the pores is between 0.05 µm and 6 µm. It could lead to a decrease in the mechanical properties of the MKGP specimens.

Figure 10 shows more information on the evolution of the tested specimens’ pore structure. MIP results showed that the MKGP concrete contains dual pore peaks (the big pores of 10 μm and the gel pores of 20 nm), while the OPC concrete shows only one pore peak (the capillary/gel pore peak of 100 nm). It shows that most of the pore sizes in the OPC specimens are distributed between 0.35 µm and 32 µm, whereas the majority of the pore sizes in the MKGP specimens are between 0.06 µm and 32 µm. Peaks corresponding to the MKGP specimens appear in the pores between 1 µm and 32 µm, of which the MKGP3 shows the highest peak compared to the other specimens. Results revealed that the MKGP3 exhibits a dominant pore size distribution of less than 7 µm. With a decrease in the coarse aggregate size, a less porous structure emerges in the MKGP1.

**Figure 11:**
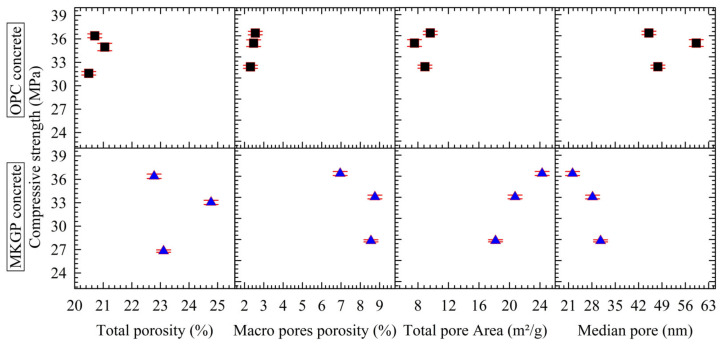
Pore structure characterization of the OPC and MKGP specimens.

The specimens tested by MIP in this study contain pores with distinct size ranges, one being micropores (<1 µm) and the other being macropores (>1 µm). Figure 11 presents the effects of the pore structure on the compressive strength of the OPC concrete and MKGP concrete. It reports the total porosity, macropore porosity, total pore area, and average (median) pore diameter that were determined from the MIP test. The average pore size can be calculated as the ratio of pore volume multiplied by four to the pore area (4V/A).

Noticeable pore structure change could be observed from the presented data. Unlike the average pore results, the total porosity, macropore porosity, and total pore area in MKGP specimens are higher than in the OPC specimens. It shows that the pore structure significantly resulted in a change of the mechanical properties of the MKGP specimens.

The mercury intrusion porosities of OPC1, OPC2, OPC3, MKGP1, MKGP2, and MKGP3 are 20.49%, 21.05%, 20.70%, 22.78%, 24.77%, and 23.11%, respectively. Although the results show that the effect of the coarse aggregate size is insignificant on the total porosity of the specimens, the MKGP concrete specimens’ porosities are higher than the OPC concrete specimens. For the MKGP concrete specimens, the macropore porosity results indicated that the larger coarse aggregate size resulted in a larger macropore porosity compared to OPC concrete, which consequently could lead to a decreased compressive strength of the MKGP concrete specimens.

Figure 11 shows that the variation of the total pore area in the OPC specimens is not remarkable, while the average pore size exhibited an increasing and decreasing tendency with an increase in the coarse aggregate size. Although the total pore area of OPC2 is smaller than that of OPC3, the average pore size of OPC2 is larger than the OPC3, which resulted in a slightly smaller compressive strength. For the MKGP specimens, however, with the increase of the coarse aggregate size, the total incoming mercury decreased, while the average pore size increased, which subsequently resulted in a decreased compressive strength. The results show that the compressive strengths of the MKGP specimens tend to decrease as the average size of pores increase. The average pore diameter of the tested specimens, according to the coarse aggregate size and type of concrete, appear in the following order: OPC2 > OPC1 > OPC3 > MKGP3 > MKGP2 > MKGP1.

## 4. Further Discussion

**Figure 12:**
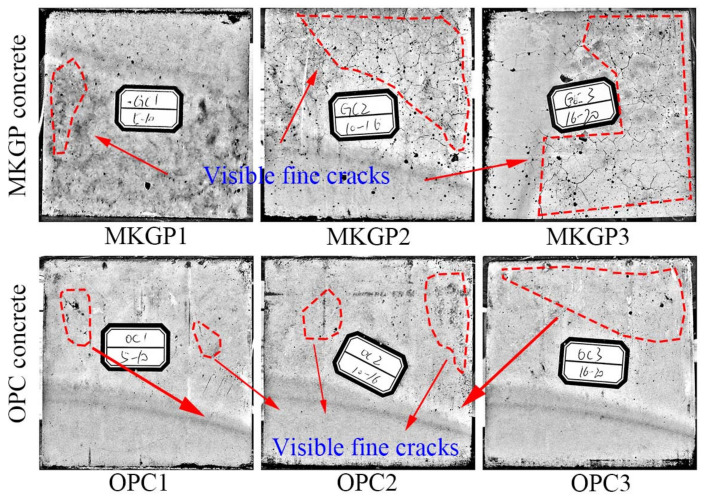
Surface cracks of the specimens before the compression test.

Compared with OPC concrete cubes, a visual inspection of the MKGP concrete cubes’ surface before the compression test showed more visible and finer cracks, as shown in Figure 12. It was associated with early surface drying. It was observed that an increase in the size of the coarse aggregate in MKGP concrete specimens increased the surface cracks after hardening. Moreover, it is known that the content of water in the MKGP concrete mixture consisted of water in the activators and added water. Therefore, the increased surface cracking in larger coarse aggregate size specimens could be related to the concrete shrinking due to the excess water that evaporates out of the MKGP specimens. Under uniaxial compression loading, as the applied load increased, small cracks appeared and propagated almost parallel to the direction of the applied load. In this situation, the weak bonding between the coarse aggregate and paste could yield interfacial cracks, as reported by Van Mier [46]. These cracks could develop and result in lateral tension. However, the findings after the compression test showed that cracks mostly occurred in the MKGP paste, which could be attributed to its higher shrinkage compared to OPC concrete.

Moreover, the central core of the OPC concrete cubes was relatively undamaged compared to the MKGP specimens. As the size of the coarse aggregate increases in the OPC concrete, the continuous crack growth could be delayed due to the confinement stresses because of the friction between the platens of the testing machine and the concrete cubes [47]. It can therefore be concluded that in the MKGP concrete specimens with a coarse aggregate of larger size, the confinement could not restrain the crack growth, as it yields lower strength compared to the smaller size of the coarse aggregate. It may be because of the high shrinkage of the MKGP concrete due to the large surface area of metakaolin [48]. 

The coarse aggregate could significantly influence the characteristics of concrete, as it occupies 70–80% of the concrete volume [49]. It is reported that in the OPC concrete of the large coarse aggregate, the increasing volume of the coarse aggregate will result in a decrease in concrete shrinkage. [49]. It is reported that the larger coarse aggregate restrains the inner strains and prevents the transition of microcracks into macrocracks [50] and hence decreases shrinkage. Consequently, the compressive strength of concrete increases with the increase of the coarse aggregate size.

SEM observations confirmed the non-uniform microstructure of the MKGP paste compared to that of the OPC concrete. It may be because, in geopolymer production, the water is not combined directly with the gel product; accordingly, a small amount of the water remains as interstitial water in the gel [51]. Moreover, a large amount of water demands to mix the metakaolin, which yields a large excess of free water. It could result in a porous microstructure due to the evaporation under ambient temperature conditions with low relative humidity [52], which yields an extensive shrinkage of the specimen [53]. Besides, the water absorption increases as a result of increasing the Si/Al ratio and decrease in the rate of the geopolymerization process, which, in turn, affects the MKGP concrete and leads to a porous and less dense microstructure and ITZ area. Therefore, the results of the previous studies, including the findings from Mastali, Kinnunen, Dalvand, Mohammadi Firouz and Illikainen [48], confirm that the MKGP paste tended toward a high shrinkage. Thus, it can be concluded that the MKGP specimens exhibit lower strength due to higher shrinkage. 

Table 6 shows the obtained values of the drying shrinkage from the previous studies, whereas, for the MKGP paste, the results are larger than for the OPC paste. It could be attributed to the effect of the large surface area and particle shape of metakaolin, and subsequently, its influence on the physical and mechanical properties. 

The observed pore structure measurements change in MKGP concrete could be attributed to its higher shrinkage, which yielded a porous structure in the MKGP specimens. The study of Yang, Zhu and Zhang [55] on geopolymers reported an enhanced relationship between the shrinkage and their micropore structure. Moreover, the lower compressive strength of the MKGP specimens can be related to the increased shrinkage [58].

**Figure 13:**
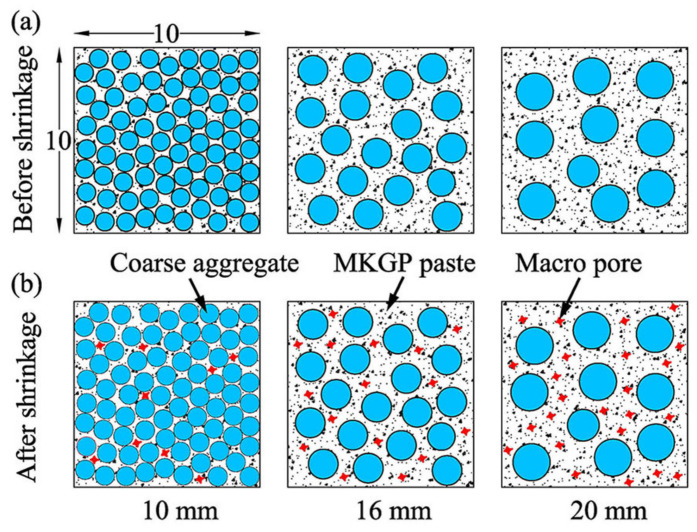
Schematic of MKGP specimens. (**a**) Before shrinkage; (**b**) after shrinkage.

Figure 13 illustrates the effect of shrinkage on the MKGP specimens. A larger MKGP paste volume appears around the coarse aggregate as the size of the coarse aggregate increases. The larger MKGP paste volume yields higher shrinkage due to its porous microstructure. Consequently, higher shrinkage results in a larger number of macropores (>1 µm), as shown in Figure 13b. As a result, it leads to a decrease in compressive strength.

## 5. Conclusions

This study investigates the effect of the coarse aggregate size on the mechanical properties of the MKGP concrete and OPC concrete. The compressive strength and splitting tensile strength of the specimens were determined after 28 days and were analyzed. From the results of the experimental work conducted in this study, the following conclusions can be drawn:

(1) The compressive strength of MKGP concrete and OPC concrete decreased and increased, respectively, as the size of the coarse aggregate increased. This behavior can be explained by the higher shrinkage of the MKGP concrete.
(2) The maximum reduction and increment in compressive strength due to the increase of the size of the coarse aggregate of OPC concrete and MKGP concrete were 13% and 36%, respectively.
(3) The splitting tensile strength decreased in both groups because coarse aggregates with larger sizes yield higher microcracks in the vicinity of the coarse aggregate.
(4) The maximum reduction in the splitting tensile strength due to the increase of the size of the coarse aggregate of OPC concrete and MKGP concrete were 28% and 114%, respectively.
(5) SEM/EDS investigations revealed that the size of the coarse aggregate does not significantly affect the ITZ area in this study. MIP results showed a larger pore diameter with increasing size of the coarse aggregate. The role of paste between coarse aggregates is therefore more pronounced as the size of the coarse aggregate increases.
(6) It can be concluded that the changes in pore structure with the size of the coarse aggregate significantly influences the strength development of the tested specimens. An increase in the size of the coarse aggregate results in a higher shrinkage of MKGP concrete and subsequently results in a larger number of macropores. Hence, the influence of macropores is significant.

## Figures and Tables

**Table 1:** Chemical composition of OPC and MK (wt.%).

Chemicals	SiO_2_	Al_2_O_3_	Fe_2_O_3_	CaO	MgO	SO_3_	K_2_O	TiO_2_	Na_2_O	LOI *
OPC	23.81	10.79	3.36	50.58	5.31	2.75	0.92	0.73	0.61	1.14
MK	53.29	41.64	0.53	1.09	0.28	-	0.14	1.13	0.07	1.83

* LOI: loss on ignition.

**Table 2:** Oxide composition of sodium silicate solution (WG).

Oxide	SiO_2_	Na_2_O	H_2_O
Mass content (%)	26.00	8.20	65.80

**Table 3:** Chemical composition of OPC and MK (wt.%).

Group	MK	WG		Cement	Coarse Aggregate	Sand	Water
		SiO_2_	Na_2_O				
OPC	-	-	-	487	1115	560	208
MKGP	268	92.82	106.77	-	1115	560	255

**Table 4:** Chemical composition of OPC and MK (wt.%).

Series		Specimens	Coarse Aggregate Size (mm)	$f_{cu}\;(\mathrm{MPa})$			$f_{t}\;(\mathrm{MPa})$		
				Indiv. *	Ave. *	SD	Indiv. *	Ave. *	SD
OPC	OPC1	OPC-1-1	5–10	31.80	31.59	0.22	4.82	4.77	0.05
		OPC-1-2		31.36			4.72		
		OPC-1-3		31.62			4.77		
	OPC2	OPC-2-1	10–16	34.53	34.96	0.48	4.08	4.14	0.08
		OPC-2-2		34.87			4.11		
		OPC-2-3		35.47			4.23		
	OPC3	OPC-3-1	16–20	36.29	36.40	0.25	3.82	3.74	0.09
		OPC-3-2		36.22			3.65		
		OPC-3-3		36.68			3.75		
MKGP	MKGP1	MKGP-1-1	5–10	36.11	36.39	0.29	3.00	3.13	0.15
		MKGP-1-2		36.68			3.29		
		MKGP-1-3		36.38			3.10		
	MKGP2	MKGP-2-1	10–16	33.27	33.05	0.27	2.69	2.85	0.15
		MKGP-2-2		32.75			2.88		
		MKGP-2-3		33.12			2.98		
	MKGP3	MKGP-3-1	16–20	26.95	26.81	0.15	1.44	1.46	0.03
		MKGP-3-2		26.65			1.45		
		MKGP-3-3		26.82			1.49		

* Indiv.: individual; Ave.: average.2.3. Mechanical Test.

**Table 5:** Comparison of theoretical and experimental results of compressive strength.

**Specimens**	Calibrated Model ($f_{c}$) (mm)	Proposed Model ($f_{tg}$) (mm)	$f_{cu}$ $/f_{c}$	$f_{t}$ $/f_{tg}$
MKGP-1-1	35.97	3.44	1.00	0.87
MKGP-1-2	35.97	3.57	0.98	0.92
MKGP-1-3	35.97	3.5	0.99	0.89
MKGP-2-1	30.85	2.83	0.93	0.95
MKGP-2-2	30.85	2.73	0.94	1.05
MKGP-2-3	30.85	2.8	0.93	1.06
MKGP-3-1	27.52	1.71	1.02	0.84
MKGP-3-2	27.52	1.67	1.03	0.87
MKGP-3-3	27.52	1.7	1.03	0.88
Average			0.98	0.93
SD			0.041	0.077

**Table 6:** Values of drying shrinkage were reported in previous studies.

OPC paste drying shrinkage (×10^−6^)		MKGP paste drying shrinkage (×10^−6^)	
Bakharev et al. [54]	600	Yang, et al. [55]	5976
Neupane [56]	550	Xiang, et al. [57]	2505

## Data Availability

All data, models, or codes that were generated or used during the study are included in this manuscript.
